# From Stem Cell to Embryo without Centrioles

**DOI:** 10.1016/j.cub.2007.07.060

**Published:** 2007-09-04

**Authors:** Naomi R. Stevens, Alexandre A.S.F. Raposo, Renata Basto, Daniel St Johnston, Jordan W. Raff

**Affiliations:** 1 The Gurdon Institute, Tennis Court Road, Cambridge CB2 1QN, United Kingdom

**Keywords:** CELLBIO, DNA

## Abstract

Centrosome asymmetry plays a key role in ensuring the asymmetric division of *Drosophila* neural stem cells (neuroblasts [NBs]) and male germline stem cells (GSCs) [1–3]. In both cases, one centrosome is anchored close to a specific cortical region during interphase, thus defining the orientation of the spindle during the ensuing mitosis. To test whether asymmetric centrosome behavior is a general feature of stem cells, we have studied female GSCs, which divide asymmetrically, producing another GSC and a cystoblast. The cystoblast then divides and matures into an oocyte, a process in which centrosomes exhibit a series of complex behaviors proposed to play a crucial role in oogenesis [4–6]. We show that the interphase centrosome does not define spindle orientation in female GSCs and that *DSas-4* mutant GSCs [7], lacking centrioles and centrosomes, invariably divide asymmetrically to produce cystoblasts that proceed normally through oogenesis—remarkably, oocyte specification, microtubule organization, and mRNA localization are all unperturbed. Mature oocytes can be fertilized, but embryos that cannot support centriole replication arrest very early in development. Thus, centrosomes are dispensable for oogenesis but essential for early embryogenesis. These results reveal that asymmetric centrosome behavior is not an essential feature of stem cell divisions.

## Results and Discussion

### The Majority of *DSas-4* Mutant Ovarian Cysts Lack Centrioles

**Figure 1:**
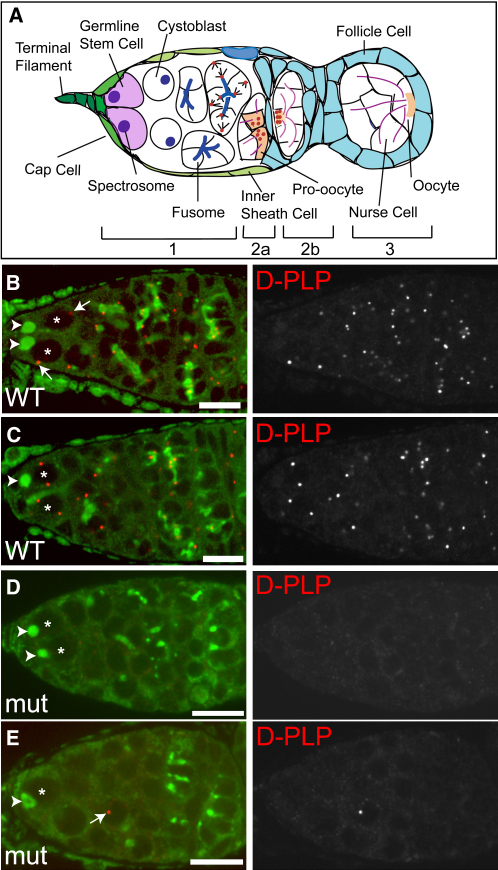
Centrosomes Do Not Segregate Asymmetrically in Female GSC Divisions (A) Schematic diagram of a *Drosophila* germarium. (B–E) WT (B and C) and *DSas-4* mutant (D and E) germaria stained for centrioles (D-PLP [red]) and the spectrosome/fusome (Shot [green]). The nuclei of the GSCs are labeled with an asterisk. In the WT GSCs, neither the single centrosomes (arrows in B) nor the replicated centrosomes (C) adopt a consistent position relative to the spectrosome (arrowheads in B–E) or the stem cell niche. The mutant germarium in (D) has no centrosomes. The germarium in (E) has only a single centrosome (arrow), which is not in a GSC. This demonstrates that the mother centrosome is not selectively retained in female GSCs. Scale bars represent 10 μm.

The *Drosophila* ovary consists of 16–20 ovarioles, chains of egg chambers proceeding through the 14 stage maturation process that begins in the germarium, at the anterior tip of the ovariole [8] (Figure 1A). In region 1 of the germarium, two to three germline stem cells (GSCs) are found in a niche composed of terminal-filament, cap, and inner-sheath cells [9]. Female GSCs divide asymmetrically to produce another GSC that remains in the niche and a cystoblast that is displaced away. This asymmetry requires that the spindle be correctly oriented with one spindle pole anchored at the spectrosome, a membranous structure found close to the niche [10].

To investigate the role of centrosomes in female GSC divisions, we have examined oogenesis in flies that lack DSas-4, a protein required for centriole replication [7]. *DSas-4* mutant flies, although morphologically normal, are uncoordinated because of the lack of cilia in their sensory neurons, and they die shortly after eclosion because they get stuck in their food. We therefore transferred mutant pupae to Petri dishes and manually fed the flies with sugar solution for 1 to 4 days before dissecting the ovaries. Because of lack of dietary protein, the mutant ovaries were invariably small, but at least 50% contained mature stage 14 eggs, indicating that oogenesis could proceed normally. In addition, we recombined the *DSas-4* mutation onto an FRT chromosome and used the FLP-FRT system to generate germline clone mutant ovaries in otherwise wild-type (WT) females [11]. We obtained similar results from both mutant ovaries and germline clone mutant ovaries. Mutant ovaries are used unless otherwise stated.

*DSas-4* mutants start to lose centrioles during embryogenesis, and no centrioles are detectable in third-instar larval brain cells [7]. These cells, however, divide extensively during larval stages, whereas female germ cells are set aside early in development and do not divide significantly until pupal stages [8]. To test whether *DSas-4* mutant germline cells retained any centrioles, we stained WT and mutant ovaries with antibodies against the centriolar protein D-PLP and the centrosomal proteins Cnn or Polo (Figure 1; data not shown). WT germaria contained many hundreds of centrioles and centrosomes (Figures 1B and 1C), whereas mutant germaria contained, at most, only a few (Figures 1D and 1E). We counted centriole numbers in mutant stem cells and found that more than 80% (n = 114) contained no detectable centrioles. Thus, although some centrioles can persist into adulthood in *DSas-4* mutant ovaries, the vast majority of cysts contain no detectable centrioles or centrosomes.

### Centrosomes Do Not Segregate Asymmetrically in Female GSC Division

The centriole pair at the core of each centrosome consists of a younger daughter and an older mother. After centrosome duplication, one centrosome will inherit the original mother centriole and the other the original daughter. During the asymmetric divisions of *Drosophila* male GSCs [2] and larval neuroblasts (NBs) [1, 3] the two centrosomes behave asymmetrically, and in male GSCs, it has been shown that a centrosome's behavior depends on its mother-daughter identity. In both cases, one centrosome (the mother in male GSCs) is anchored close to a specific cortical region during interphase (near the stem cell niche for GSCs, and close to apical polarity cues for NBs), whereas the second is mobile. As the cells enter mitosis, the second centrosome localizes to the side of the cell opposite the anchored centrosome, ensuring correct spindle orientation with respect to either the niche or the polarity axis. In male GSCs, the mother centriole is thus always retained in the GSC [2], and it has been proposed that differential centrosome inheritance might be an essential feature of stem cell divisions [12]. Moreover, in NBs that lack centrioles and centrosomes [7], and in male GSCs that lack functional centrosomes [13], asymmetric division is partially perturbed.

We found that in WT female GSCs, the interphase centrosome did not adopt a consistent position with respect to either the niche-GSC interface or the spectrosome (arrows, Figure 1B). Even after centrosome duplication, the two centrosomes appeared to be randomly positioned within the cell (Figure 1C). It was not until the mitotic spindle had fully formed that we observed a consistent orientation of the centrosomes relative to the spectrosome and niche (see below). Although live imaging would be required to fully describe centrosome movements in female GSCs, it appears that the centrosomes do not behave in the manner observed in male GSCs and NBs.

Examining the distribution of the few remaining centrioles in *DSas-4* mutant germaria allowed us to test whether the mother centriole was always retained in the GSC after division. If this were the case, then the failure of centriole replication in a GSC should result in the production of a GSC with one mother centriole and an acentriolar cystoblast; we would never expect to see a mutant germarium in which all of the stem cells lacked centrioles while the cystoblasts or cyst cells contained them. An example of such a germarium is shown in Figure 1E, suggesting that the mother centriole cannot always be retained in the female GSC.

### *Drosophila* Female Germline Stem Cells Divide Asymmetrically without Centrosomes

**Figure 2:**
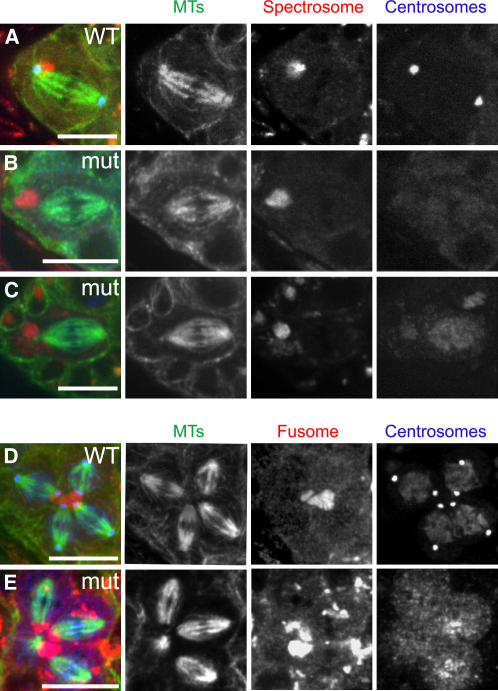
Centrosomes Are Not Required for Spindle Orientation in Female GSCs or Cysts (A–C) WT (A) and *DSas-4* mutant (B and C) female GSCs showing the attachment of one spindle pole to the spectrosome (Shot [red]). Centrosomes, revealed by Cnn (A and B) or Polo (C) staining (blue), are absent in the mutant GSCs. MTs are shown in green. Anterior is to the left. (D–E) WT (D) and *DSas-4* mutant (E) four-cell cysts. Despite the absence of centrosomes (revealed by Polo staining in blue) in the mutant cyst, the four spindles (one of which is only partially visible in this Z section) are clearly attached to the fusome. Scale bars represent 10 μm.

These results strongly suggested that asymmetric centrosome inheritance does not occur in female GSC division. To investigate whether centrosomes are nevertheless required for spindle orientation, we examined mitotic stem cells with fully formed spindles (Figures 2A–2C). In WT stem cells, one spindle pole was always associated with the spectrosome, and the spindle was always positioned so that the cell would divide asymmetrically with respect to the niche, i.e., one daughter would remain in the niche and the other would be displaced away (12/12 cells; Figure 2A). Similarly, in *DSas-4* mutant stem cells, one of the acentrosomal spindle poles was always associated with the spectrosome, and the spindle was always positioned such that the cell would divide asymmetrically with respect to the niche (13/13 cells; Figures 2B and 2C).

The displaced cystoblast undergoes four rounds of division, producing a cyst of 16 interconnected cells. During these divisions, one pole of each spindle is anchored to the fusome, a branched structure closely related to the spectrosome [14], and this interaction has been proposed to involve the astral microtubules (MTs) emanating from the centrosome [4]. We confirmed that in WT mitotic cysts, one pole of each spindle was always associated with the fusome (n = 14 cysts, 49 mitotic cells; Figure 2D and Figure S1 in the Supplemental Data available online) and found that this was also true for the acentrosomal spindle poles in *DSas-4* mutant cysts (n = 27 cysts, 117 mitotic cells; Figure 2E and Figure S1). We conclude that centrosomes are not required for the proper orientation of the spindle relative to the spectrosome or niche in female GSCs or for the proper orientation of the spindles relative to the fusome in mitotic cysts.

### The Oocyte Is Correctly Specified in the Absence of Centrioles

One of the 16 cells differentiates into the oocyte, whereas the others become nurse cells. Centrosome asymmetry has been proposed as one means of selecting the oocyte because only one of the 16 cells inherits the original mother centriole from the single-cell cystoblast [5]. The centrioles from the nurse cells then migrate into the oocyte [15], moving to the posterior, where they colocalize with the major microtubule-organizing center (MTOC). The MTOC nucleates a MT network extending into the nurse cells, and this is required for the localization of oocyte specific proteins [16].

**Figure 3:**
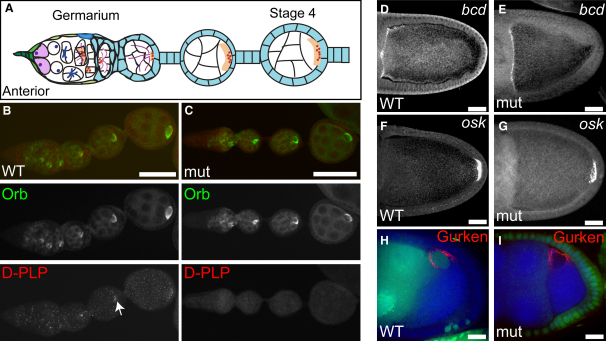
The Oocyte Is Correctly Specified and mRNAs Are Properly Localized in the Absence of Centrioles (A) Schematic diagram of Orb localization (light orange) in a *Drosophila* ovariole. (B and C) WT (B) and *DSas-4* mutant (C) ovarioles showing the accumulation of Orb protein (green) in the oocyte of each cyst. D-PLP staining (red) shows the presence of many centrioles in the WT ovariole, and these can be seen to accumulate in the oocyte (arrow). No centrioles are detectable in the mutant ovariole. Scale bars represent 50 μm. (D–G) Stage 10A oocytes showing the localization of *bicoid* (*bcd*) mRNA to the anterior margin (D and E) and *oskar* (*osk*) mRNA to the posterior pole (F and G). The localization of both mRNAs is indistinguishable in WT oocytes (D and F) and *DSas-4* mutant germline clones (E and G). Scale bars represent 25 μm. (H and I) Stage 8 egg chambers showing the localization of Gurken protein (red) between the nucleus and the anterior-dorsal corner of the oocyte. Gurken localizes identically in WT (H) and in a *DSas-4* mutant germline clone (I) (marked by the absence of nuclear GFP in green). DNA is in blue. Scale bars represent 20 μm.

To investigate oocyte specification in the absence of centrioles, we examined the localization of Orb in WT and mutant oocytes; this protein normally accumulates at the posterior of the oocyte, thus providing a readout of both oocyte specification and polarity [17] (Figure 3A). The accumulation and localization of Orb was indistinguishable in WT and *DSas-4* mutant oocytes (Figures 3B and 3C; n > 100 ovarioles). The same was also true in WT and *DSas-4* mutant ovarioles stained with Polo antibodies (n > 200) because Polo also normally accumulates in the early oocyte (data not shown). We conclude that the specification of the oocyte and the initial establishment of oocyte polarity occur normally in the absence of centrioles.

### A Centrosome Is Not Required for Microtubule Reorganization and mRNA Localization during Midoogenesis

In *Drosophila*, the anterior-posterior (A-P) and dorsal-ventral axes are defined by the localization of three mRNAs to distinct positions within the oocyte: *bicoid* (*bcd*) to the anterior cortex, *oskar* (*osk*) to the posterior cortex, and *gurken* (*grk*) to a cap over the nucleus in the anterior-dorsal corner. The proper localization of these mRNAs depends on a dramatic reorganization of the MT cytoskeleton during midoogenesis: The posterior MTOC is disassembled, most of the centrioles disappear, and the nucleus migrates to the anterior of the oocyte [18]. Interestingly, a single centriole-containing centrosome has recently been found to be closely associated with the nucleus at these stages, and it has been suggested that this centrosome-nucleus complex acts as the major MTOC during midoogenesis [6]. Surprisingly, we found that the MT cytoskeleton was indistinguishable in WT and mutant oocytes (Figure S2). MTs were concentrated at the posterior of the nucleus in 18/18 stage 6 mutant oocytes. The nucleus migrated normally to the anterior in 24/24 stage 7 mutant oocytes, and the MT cytoskeleton had repolarized, exhibiting its characteristic A-P gradient in 20/20 stage 9 mutant oocytes. The localization of *bcd* (n = 14) and *osk* mRNAs (n = 24) and Gurken protein (n = 24) was also indistinguishable in WT and mutant oocytes (Figures 3D–3I). We conclude that centrioles and centrosomes are not essential for MT reorganization or mRNA localization during midoogenesis.

### Centrioles Are Essential for Early Embryonic Development

Because *DSas-4* mutant flies cannot mate to produce progeny [7], it has not previously been possible to examine whether centrioles are essential for the very rapid mitotic divisions in the early syncytial embryo. We therefore used the FRT *DSas-4* chromosome to generate homozygous germline clone embryos lacking the maternal contribution of DSas-4.

**Figure 4:**
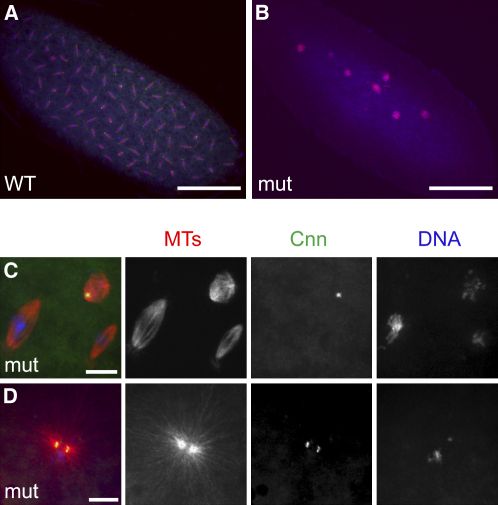
Centrioles Are Essential for Early Embryonic Development (A and B) WT (A) and *DSas-4* mutant (B) embryos from a 1–4 hr collection stained for tubulin (red), centrosomes (Cnn [green]) and DNA (blue). The WT embryo is ∼1 hr old and contains many well-organized spindles. The mutant embryo, although at least 1 hr old, is arrested early in development with only 6–7 disorganized anastral spindles. Scale bars represent 100 μm. (C) High magnification view of spindles in a *DSas-4* mutant embryo. All the spindles appear to be anastral, although one possesses a Cnn dot at one pole. This embryo contained a second Cnn dot not shown here. Scale bars represent 10 μm. (D) A *DSas-4* mutant embryo containing two closely associated Cnn dots that each nucleate a large MT aster. Scale bars represent 10 μm.

An initial examination revealed that these embryos never hatched as larvae (>500 embryos scored). To investigate when they failed in development, we fixed timed collections and stained them for DNA, tubulin, and the centrosomal marker Cnn (Figure 4). We found that all the embryos arrested very early in development with few nuclei (usually only one to eight). The chromatin was almost always in a mitotic state and was usually associated with anastral spindles (Figures 4B and 4C). Surprisingly, however, we often observed two Cnn-containing structures in these embryos, and these were often associated with astral MTs, suggesting that they were centrosomes (Figures 4C and 4D). Thus it appears that the single sperm centriole is capable of going through at least one round of replication in embryos lacking DSas-4 protein. We observed a very similar phenotype of early arrest with two centrosomes in germline clone embryos lacking maternal DSas-6 (data not shown), another protein required for centriole replication [19]. We therefore conclude that centrioles are essential for early embryonic development in *Drosophila*.

It has been shown that flies homozygous for mutations that disrupt centrosome function, such as *cnn* [20, 21] and *d-tacc* [22], although viable, lay embryos that accumulate mitotic errors and die. In these mutants, however, the embryos only start to accumulate defects later in embryogenesis, when the nuclear density increases and the spindles start to collide. We speculate that the very early arrest of *DSas-4* mutant embryos might be because centrosomes are required to catalyze the efficient destruction of Cyclin B on the mitotic spindle in early embryos [23]. The destruction of Cyclin B at the end of mitosis is initiated at centrosomes in *Drosophila* embryos [24], and, if centrosomes detach from the spindle, Cyclin B is not properly destroyed and the spindles arrest in mitosis [25]. This arrest appears to be very similar to what we observe in *DSas-4* mutant embryos.

## Conclusions

By studying the role of the centriole from stem cell to embryo, we have shown it to be essential for embryogenesis but dispensable for asymmetric female GSC division and oogenesis. Thus, asymmetric centrosome behavior is not an essential feature of stem cell division. Instead, different types of stem cells can use different mechanisms for ensuring the proper alignment of the mitotic spindle during cell division. Given the evidence that faulty spindle alignment can contribute to tumorigenesis [26], there would be considerable evolutionary pressure for stem cells to optimize their spindle orientation mechanism to their particular circumstances.

## Experimental Procedures

### Fly Stocks

Mutant alleles used were *DSas-4^S2214^* [7] and *DSas-6^c02901^* [19]. We selected the mutants as nontubby pupae from a *DSas-4^S2214^* /Tm6C stock, transferred them to Petri dishes, and fed the flies with sugar solution for 1 to 4 days before dissecting the ovaries. *w^67^* flies were used as controls.

### Germline Clones

Germline clones were made with the FLP-FRT technique [11] after the recombination of the mutations with *FRT82B* and were identified by the absence of nlsGFP or with the FLP-DFS system [27]. Clones were induced by heat shocking third-instar larvae at 37°C for 2 hr on three consecutive days. Ovaries were dissected 14 days after eclosion, and embryos were collected up to 36 days after heat shock.

### Analysis of Ovaries and Embryos

Ovaries were fixed in 10% paraformaldehyde (PFA) for 8 min or, to preserve spindles, dissected, fixed, and stained as described in [28]. GSCs were identified by their position adjacent to the cap cells and by the spherical spectrosome at the anterior of the cell. We performed *osk* and *bcd* RNA in situ hybridization as described in [29]. Embryos from 0–3 hr or 1–4 hr collections were processed as described previously [30]. Slides were analyzed with a Perkin Elmer ERS Spinning Disc confocal system.

### Antibodies

The following antibodies were used: rabbit anti-D-PLP (1:1000) [30]; mouse DM1a monoclonal anti-α-tubulin (1:1000) (Sigma); mouse anti-Lamin A (1:250) [31]; rat anti-Gurken (1:1000) (T. Schupbach); rabbit anti-Centrosomin (1:1000) (R.B., unpublished data); guinea-pig anti-Shot spectrin repeat (1:1000) [32]; and rabbit anti-phospho-PLK Ser137 (anti-Polo) (1:400) (Cell Signaling Technology). Mouse anti-Orb 4H8 and 6H4 (1:200) each (developed by P. Schedl) were obtained from the Developmental Studies Hybridoma Bank developed under the auspices of the National Institute of Child Health and Human Development (NICHD) and maintained by The University of Iowa, Department of Biological Sciences, Iowa City, IA. Alexa 488, Cy3, and Cy5 secondaries were from Molecular Probes or Jackson Laboratories.

## Supplemental Data

Document S1. Two Figures
