# Supplementary material for: From Stem Cell to Embryo without Centrioles
Source: Curr Biol. 2007 Sep 4;17(17):1498–503. doi: 10.1016/j.cub.2007.07.060 (PMC1971134; doi:10.1016/j.cub.2007.07.060)
Supplement: Document S1. Two Figures [file mmc1.pdf]

Supplemental Data

From Stem Cell to Embryo without Centrioles

Naomi R. Stevens, Alexandre A.S.F. Raposo,
Renata Basto, Daniel St Johnston, and Jordan W. Raff

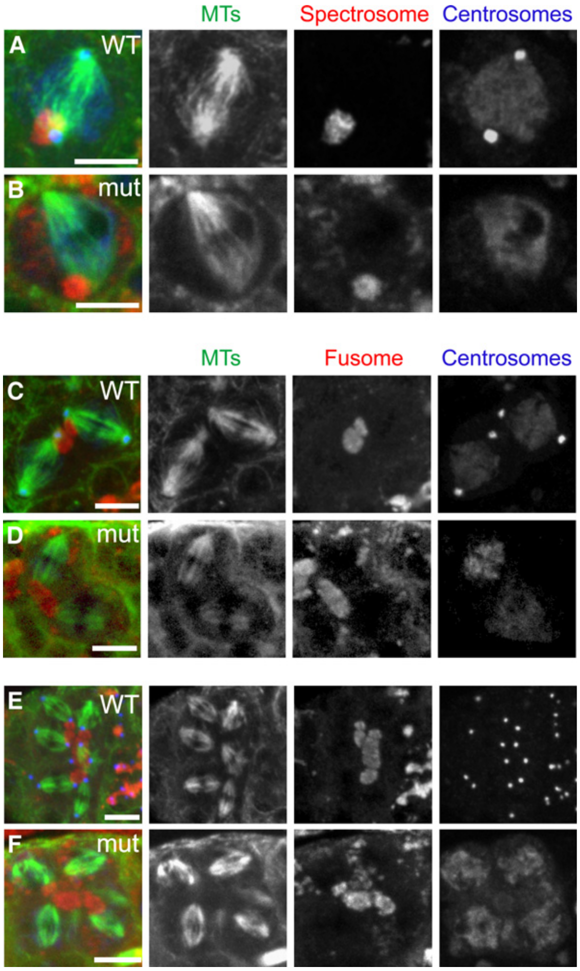

Figure S1. Centrosomes Are Not Required for Spindle Orientation during the Mitotic Divisions of Oogenesis

(A and B) WT (A) and *DSas-4* mutant (B) one-cell cystoblasts.
(C and D) WT (C) and *DSas-4* mutant (D) two-cell cysts.
(E and F) WT (E) and *DSas-4* mutant (F) eight cell cysts.
In all cases, one pole of each spindle (stained for tubulin [green]) is attached to the fusome (Shot [red]). Centrosomes (blue) are revealed by staining for Polo (A–D and F) or D-PLP (E). Scale bars represent 5 μm.

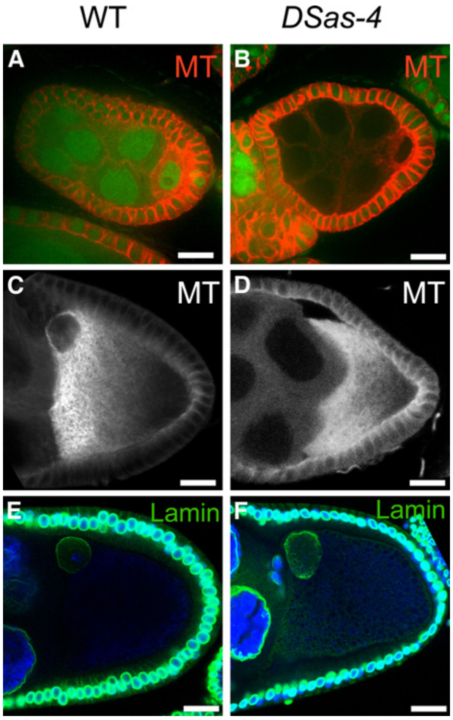

Figure S2. A Centrosome Is Not Required for Microtubule Reorganization or Nuclear Migration during Midoogenesis

(A and B) Stage 6 egg chambers with MTs (tubulin [red]) enriched at the posterior of the oocyte in both the WT (A) and a *DSas-4* mutant germline clone (B) (marked by the absence of GFP in green).
(C and D) Stage 9 oocytes showing an indistinguishable anterior-to-posterior gradient of MTs in the WT (C) and a *DSas-4* mutant germline clone (D).
(E and F) Stage 9 oocytes showing that the nucleus (lamin A [green]) migrates to the anterior in both the WT (E) and *DSas-4* mutant germline clones (F). Nuclear morphology varies within a similar range in WT and *DSas-4* mutant oocytes. DNA is in blue.
Scale bars represent 20 μm.
